# A needs assessment for faculty development at two medical colleges of Dow University of Health Sciences, Karachi

**DOI:** 10.12669/pjms.346.16302

**Published:** 2018

**Authors:** Nusrat Shah, Afifa Tabassum, Nighat Shah

**Affiliations:** 1 *Dr. Nusrat Shah, MBBS, MCPS, FCPS, MHPE, Dow University of Health Sciences, Karachi, Pakistan*; 2 *Dr. Afifa Tabassum, MBBS, MHPE, Liaquat National Hospital Medical College, Karachi, Pakistan*; 3 *Dr. Nighat Shah, MBBS, FCPS, MRCOG, MHPE, Jinnah Sindh Medical University, Karachi, Pakistan*

**Keywords:** Faculty development, Medical education, Needs assessment

## Abstract

**Objective:**

To identify the perceptions of the faculty about their current level of pedagogical skills and their desire to improve these skills in future.

**Methods:**

This cross-sectional study was conducted at two medical colleges of Dow University of Health Sciences, Karachi from March 1, 2015 to April 30, 2015. A re-validated questionnaire was used comprising three parts; 1) Demographic details, 2) Fifteen educational domains each having 2 statements, first indicating minimum knowledge of domain and second showing maximum. Each statement had two 9-point anchored scales, first indicating current knowledge and second, desired knowledge of the faculty. 3) Factors which are important considerations for attending educational workshops. The data was analyzed by statistical software SPSS 17.

**Results:**

The response rate was 54%. The mean age of faculty was 43.42 ± 8.41, largest proportion being assistant professors (85; 47%). For all educational domains, faculty perceived their knowledge to be moderately high (Mean = 5.9 +/- 1.8 to 6.8 +/- 1.7 and Median: 6 to 7). However, they desired to improve their pedagogical skills further in all domains (Mean = 8.2 +/- 1.1 to 8.4 +/- 1.1, Median: 9), p-values < 0.001. Nearly 95% teachers are willing to attend faculty development workshops.

**Conclusion:**

Our faculty perceives their current pedagogical skills to be moderately high. However, they would like to improve these skills to a higher level in all educational domains.

## INTRODUCTION

Faculty development (FD) is a crucial component of medical education today, mainly due to the rapidly changing healthcare scenario. Advances in medical and information technology, commercialization, litigation, and the vast amount of medical knowledge have led to changes in the needs and expectations of the society. Society now wants doctors to be not just medical experts, but also good communicators, collaborators, managers, scholars, professionals and health advocates. This calls for a drastic reform in medical education, which would include FD as one of the major interventions. Moreover, as models of health care change to patient-centered, inter-professional, and systems-based care, the faculty needs to be trained to produce graduates who will follow these models of care and be effective healthcare leaders for tomorrow.^1^,^2^

Traditionally, medical school faculty members are hired only for their content knowledge and skills rather than for their teaching ability. However, it is increasingly being realized now that faculty members need to be trained in teaching and learning strategies to meet the demands of today’s, newer, more complex healthcare system.^3^ FD can help the faculty realize their various expected roles and responsibilities, such as, those of a researcher, clinician, administrator, and an educational leader.^4^ It can also help the faculty realize their sense of social accountability and lead to the pursuit of excellence in medical education.^5^

FD is imperative for all medical universities. It needs to be systematic and planned with an emphasis on newer evidence-based teaching strategies e.g. work-based learning, e-learning and community-based learning.^6^ FD should be part of an overall strategic plan developed by department of medical education of an institution and should be matched to the needs of the institution and faculty, available resources and feasibility.^7^ In addition, FD is an important institutional approach towards developing teaching excellence among faculty by promoting educational infrastructure, capacity building and collaboration and expertise sharing with international colleagues.^8^

However, to be successful, FD programs should be implemented after conducting needs assessment (NA) studies. Institutions can greatly benefit from such studies by learning what particular skills are required by the faculty, so that programs can be targeted to address those needs, resulting in effective utilization of limited resources.^9^

Secondly, NA studies help teachers to realize their teaching capabilities and allow the program managers to optimize their activities. They also help to explore the key competencies to be prioritized, faculty’s willingness to participate, their time commitments and their suggestions on FD initiatives.^10^

Finally, a well conducted need assessment can also help in paying attention to the faculty’s humanistic and professional needs so that steps can be taken to enhance their sense of personal and professional fulfillment.^11^

Faculty development is a relatively new concept for medical schools of Pakistan. A few medical institutions in Pakistan are providing some training in communication skills and teaching skills, however, such trainings have not been reported to be the result of a needs assessment study.^12^

This needs assessment study was conducted to identify the perceptions of the faculty of Dow Medical College (DMC) and Dow International Medical College (DIMC) about their current level of pedagogical skills and their future needs and preferences about these skills.

## METHODS

### Context

The Dow University of Health Sciences (DUHS) has recently reformed its traditional curriculum into an integrated modular curriculum, introduced MCQs (one best type), OSPE and OSCE as assessment instruments and changed the annual system into semester system. The number of lectures has been significantly reduced and small group teaching strategies like case-based learning introduced. However, a lot of faculty development is needed to ensure smooth implementation of these strategies.

This Cross-sectional, comparative study was undertaken for a period of two months. The total number of faculty members available at the time of study was 334 (DMC: 203, DIMC: 131). The entire faculty was sent the hard copy of questionnaire through the dispatch department of DUHS. Visiting and part-time faculty members were excluded.

### The Questionnaire

A structured questionnaire, based on a validated questionnaire developed by Amin Z et al.^13^ was used after pilot testing, validating and contextualizing. We modified the questionnaire after reviewing the literature and considering areas relevant to our own context. The draft questionnaire was Pilot tested by administering it to 10 faculty members who were attending the MHPE classes with first author during contact period of MHPE course at DUHS. The questionnaire was modified after considering feedback from faculty members, reviewed again by MHPE experts, and then finalized. The questionnaire had three parts. The first part dealt with demographic information including age, gender, academic rank, teaching experience, and department. The second part had fifteen items or educational domains with a 9-point anchored scale for each item. The first statement described someone having very little knowledge of the item and the second statement referred to someone having adequate knowledge of that domain. The scale points 1 to 3 were defined as ‘limited knowledge’, 4 to 6 as ‘moderate knowledge’ and 7 to 9 as ‘substantial knowledge’. For each item, participants needed to identify their current level of knowledge and what they believed their future knowledge should be. The third section was about factors which are important considerations for attending educational workshops.

A cover letter was attached to the questionnaire which explained the purpose and importance of the study, and served as the informed consent. It assured respondents that the questionnaire was voluntary and anonymous, the information obtained would be kept strictly confidential and only the aggregate results will be reported.

### Statistical analysis

Data were entered into the statistical software SPSS-17. The descriptive data was presented in frequencies and percentages. The means with standard deviations and medians were calculated for each of the 15 items. The difference between current and desired knowledge was determined by the non-parametric Wilcoxon-Signed Rank test to see if the respondents reporting limited or modest knowledge wanted their knowledge to be in the higher level (modest or substantial). A p-value of < 0.05 was considered to be significant. Chi-square test was used to determine the difference between categorical variables, and to find whether the response to any item differed between the two colleges, between the academic ranks of the faculty or between the different teaching experiences of the faculty.

### Ethical approval

Ethical approval was taken from the Institutional Review Board (IRB) of DUHS. Ref: IRB-546/DUHS/Approval/2015/14.

## RESULTS

The total number of available faculty members was 334 (DMC: 203, DIMC: 131). Completed questionnaires were returned by 181 faculty members giving a response rate of 54% (DMC = 98/203; 48%, DIMC = 83/131; 63%).

The total percentage of teaching activities exceeds 100% as individual respondents were involved in more than one teaching activities.

### Demographics

The mean age of faculty was 43.42±8.41. The largest proportion of faculty members were Assistant Professors (47%) followed by junior faculty (25%). Majority of respondents belonged to clinical sciences (52%). The largest proportion of teachers had 4 to 9 years experience (37%) followed by those having 10 to 19 years experience (29%). Lecture was the most common teaching method in both colleges (83%).Table-I

**Table-I:** Demographic characteristics, academic rank, department and teaching experience.

	DMC (N=98) n* (%)	DMC (N=83) n* (%)
***Age***
≤ 40	29 (33)	44 (55)
41-50	33 (38)	20 (25)
> 50	26 (30)	16 (20)
***Gender***		
Male	54 (56)	31(41)
Female	42 (44)	45(59)
***Academic Rank***		
Professors	17 (18)	10 (12)
Associate Professors	14 (14)	8 (10)
Assistant Professors	48 (49)	37 (45)
Lecturers/SRs	18 (18)	28 (34)
***Department***		
Basic Sciences	24 (24)	63 (76)
Clinical Sciences	74 (76)	20 (24)
***Teaching Experience***		
≤ 3	12 (12)	17 (20)
4 – 9	28 (29)	38 (46)
10 – 19	36 (37)	17 (20)
≥ 20	22 (22)	11 (13)
***Teaching Activities***		
Lecture	80 (82)	71 (86)
Tutorials	73 (74)	63 (76)
Clinical	78 (80)	33 (40)
Lab teaching	15 (15)	41 (49)

* Total n varies due to small numbers of missing data.

The means and median points of respondents’ current knowledge and future needs for various educational domains (Cronbach’s alpha=0.968). Table-II. Generally our faculty members have reported their current knowledge either at higher end of modest level (scale points 4 to 6) or at the lower end of the “substantial” level (scale points 7 to 9). Respondents reported higher knowledge in areas such as teaching in lecture and large group, bedside and clinical teaching, teaching in tutorials and small groups, and teaching communication and counseling skill (Mean 6.6 to 6.8). However even in these areas, participants reported a need for improving these skills further (Mean above 8.0 and Median 8.0 and 9.0; p < 0.001).

**Table-II:** Means, Standard Deviations and Medians for educational domains.

Educational domains Alpha = 0.968	Current		Desired		Wilcoxon Ranks test
	
	Mean (±1 SD)	Median	Mean (±1 SD)	Median	p-value
Teaching & Learning concept	6.2 (±1.7)	7	8.3 (±1.0)	9	< 0.001
Course & Curriculum planning	5.9 (±1.8)	6	8.2 (±1.1)	9	< 0.001
Educational Objectives	6.3 (±1.7)	7	8.3 (±1.0)	9	< 0.001
Lecture & Large group teaching	6.8 (±1.7)	7	8.4 (±1.0)	9	< 0.001
Teaching in Tutorials	6.6 (±1.7)	7	8.4 (±1.0)	9	< 0.001
Teaching Communication skills	6.6 (±1.7)	7	8.4 (±1.0)	9	< 0.001
Teaching Bedside & Clinical	6.7 (±1.8)	7	8.2 (±1.1)	8	< 0.001
Facilitating CBL	6.5 (±1.8)	7	8.3 (±1.1)	9	< 0.001
Giving feedback	6.3 (±1.9)	7	8.3 (±1.0)	9	< 0.001
IT & Computer skills	6.2 (±1.7)	7	8.3 (±1.0)	9	< 0.001
Selecting Assessment Instrument	6.2 (±1.7)	7	8.3 (±1.1)	9	< 0.001
Assessment using essay & MEQs	6.1 (±1.8)	6	8.3 (±1.0)	9	< 0.001
Assessment using MCQs	6.5 (±1.7)	7	8.4 (±1.1)	9	< 0.001
Assessment using OSCEs	6.5 (±1.8)	7	8.4 (±1.1)	9	< 0.001
Assessment of Professional behavior	6.1 (±1.9)	6	8.3 (±1.2)	9	< 0.001

The two factors considered most important for attending FD workshops were “My educational needs” and “Emphasis on education” followed by “Availability of time, and “Institutional support”. Course fee was considered to be the least important factor.

The vast majority of faculty members would like to attend at least one FD workshop in current year (172; 95%), preferred the trainings to be half day rather than full day (135; 79%), and on week days rather than week-ends (105; 61.4%). The top two faculty development workshops chosen by the faculty were Research (56; 31%) and leadership in education (49; 27%).

## DISCUSSION

Our results show that the faculty perceives a positive need for training to improve their pedagogical skills. Both colleges have a predominance of junior faculty. Evidence shows that the most important asset of a medical school is its faculty and success of an institution is determined by the extent to which it invests and nurtures the career development of its most junior faculty members.^14^,^15^

Our faculty perceives their current pedagogical knowledge to be at moderate to substantial level. However, they indicated their need to improve their knowledge and skills further across all educational domains. The areas with most significant difference between current and desired knowledge of the faculty were course and curriculum planning, assessment using essay and MEQs, and assessment of professional behavior. Their current level of knowledge was lowest for these three areas (Table-II). Similar results were reported by Amin Z et al.^13^ Gaps in developing course and curriculum were also identified by Adkoli BV et al.^10^

Generally there was a significant difference between current and desired knowledge of faculty members in all educational domains (p < 0.001). This coincides with results from a study by Amin Z et al.^13^

Among the factors which faculty considers important for attending educational workshops, “My educational needs” and “Emphasis on education” were the most important. Evidence shows that most faculty members may consider themselves to be good teachers and may not realize their need for training to improve their teaching skills. Teaching is considered a ‘natural talent’ which cannot be learnt and the faculty only begins to understand the program benefits and their own educational needs once they have participated in an FD program.^16^ Institutional support and Time constraints were also considered to be important factors for attending educational workshops. Similar findings have been reported by other authors too.^16^,^17^

**Table-III:** Factors important for attending educational workshops.

	DMC (N=98) n* (%)	DMC (N=83) n* (%)
***Factors most important for attending workshops***		
Availability of time	81 (84)	59 (71)
Emphasis on education	90 (95)	67 (82)
Course fee	39 (41)	30 (36)
My educational needs	88 (93)	77 (93)
Institutional support	84 (88)	51 (62)
***Attend one FD program***		
Yes	91 (94)	80 (96)
No	6 (6)	3 (4)
***Timing of workshops***		
Half Day	71 (77)	64 (82)
Full Day	21 (23)	14 (18)
***Days of workshops***		
Week days	50 (55)	54 (68)
Week-ends	39 (43)	24 (30)

A study from Pakistan reported that developing a system of incentives and awards for recognition of excellence in teaching, professional growth and research can help in motivating faculty to attend educational workshops.^18^ Moreover, the educational environment or “culture” of an institution may have a powerful influence on FD as it shows the organization’s attitude towards the value of teaching and scholarship.^17^,^19^

The large majority of our faculty showed willingness to participate in educational programs. Most teachers indicated their preference for half-day programs conducted during week days. A national survey of faculty development in US teaching hospitals also reported that eighty percent of their workshops were half-day workshops.^19^

The two most popular workshops which faculty would like to attend were on research and leadership in education. This may be because research has remained a neglected area in medical curriculum of Pakistan. A study from Pakistan reported only about 42% of faculty members from public and private medical universities were involved in doing research.^20^ In addition, training in leadership skills is not a regular component of medical curricula worldwide. It has been suggested that such trainings should be an essential component of medical education to prepare the physicians for future leadership roles and responsibilities.^21^

### Strength and Limitations of the Study

The strength of our study is that we used a validated and reliable questionnaire which was piloted and modified according to our own context. The limitations are that the study sample may not be representative of the entire faculty of DMC and DIMC as the response rate from DMC was only 45% and included more clinical faculty compared to basic science faculty. Similarly, although response rate from DIMC was 63%, but this was mainly from basic science faculty. Secondly, we have used a questionnaire which has the inherent flaw of response and social desirability bias and is not an objective assessment of the pedagogical knowledge and skills of the faculty. Hence, our results may not be generalizable to other institutions as our findings are context-based.

The next step in assessing the needs of the faculty can be a qualitative NA study to know in-depth attitudes of faculty about FD programs and secondly to do an objective analysis of the faculty’s pedagogical knowledge and skills.

## CONCLUSION

This study has been able to bring to light faculty perceptions about their current pedagogical skills which they think are moderately high. However, they would like to improve these skills to a higher level in all educational domains. Faculty from both medical colleges and across all teaching experiences is willing to attend FD workshops.
